# Development and validation of a novel risk model in newly diagnosed *de novo* bone metastatic prostate cancer (M1b): a retrospective study

**DOI:** 10.7717/peerj.14615

**Published:** 2023-01-12

**Authors:** Yang Zhang, Junqi Wang, Li Ding, Yuxin Zheng, Chuang Wu, Kun Wang, Wentao Xia, Peng Ge

**Affiliations:** Department of Urology, the Affiliated Hospital of Xuzhou Medical University, Xuzhou, Jiangsu, China

**Keywords:** Bone metastatic prostate cancer, Risk model, M1b

## Abstract

**Background:**

Previous studies suggested that bone metastasis has a significant effect on the time of progression to metastatic castration-resistant prostate cancer (CRPC) for newly diagnosed *de novo* bone metastatic hormone-sensitive prostate cancer (mHSPC). Nevertheless, the effect of different bone metastasis sites was not fully evaluated. This study aimed to develop and validate a novel bone metastatic risk model.

**Methods:**

We enrolled 122 patients who were newly diagnosed with *de novo* bone metastatic prostate cancer following primary androgen deprivation based therapy at our institution from January 2008 to June 2021. The metastatic bone sites were classified into six sites: skull; cervical, thoracic, and lumbar vertebrae; chest (ribs and sternum); pelvis; upper limbs; and lower limbs. We calculated the bone metastatic score (BMS) for each site: 0 points were assigned for non-metastasis and 1 point was assigned for metastasis. The X-tile was adopted to acquire optimal cutoff points of BMS. We defined high-risk group (HRG) as BMS ≥ 3 and low-risk group (LRG) as BMS < 3. The new bone risk stratification was validated by calculating the area under the receiver operating characteristic curve (AUC). Subsequently, the relevant clinical prognostic variables were added to construct a predictive nomogram for predicting CRPC.

**Results:**

The median patient age was 73 years. Most patients had Gleason score ≤8 (93 cases, 76.2%). The median follow-up duration was 11.5 months (range: 2–92 months). Eighty-six patients progressed to CRPC during the follow-up. The most common bone metastatic site was the pelvis (90.2%). The median BMS was 4. Seventy-six patients had HRG, while forty-six had LRG. The 1-, 2-, and 3-year AUCs for H/LRG were 0.620, 0.754, and 0.793, respectively. The HRG was associated with earlier time to CRPC. A nomogram based on four parameters (Gleason score, H/LRG, prostate-specific antigen [PSA] nadir, and time to PSA nadir) was developed to predict CRPC. Internal validation using bootstrapping demonstrated good accuracy for predicting the CRPC (C-index: 0.727). The calibration analysis demonstrated that the model performed well.

**Conclusion:**

We established a novel H/LRG risk model for newly diagnosed *de novo* bone metastatic prostate cancer, which provided evidence to support clinical decision-making.

## Background

Prostate cancer is one of the major causes of morbidity and mortality in men worldwide. There will be an estimated 268,490 new cases of prostate cancer in the United States in 2022, which will account for 27% of all new cancer cases in men; approximately 34,500 patients will die due to prostate cancer, significant proportions of whom are in the metastatic stage (mainly bone metastases) (Siegel et al., 2022).

Metastatic prostate cancer is considered incurable and is often treated with palliative therapies. Most metastatic prostate cancers will respond initially to therapies that interfere with the androgen receptor (AR) signaling axis (Huggins & Hodges, 2002). Therefore, the main component of the current standard of care for patients with metastatic prostate cancer is androgen deprivation therapy (ADT). ADT consists of systemic treatments that reduce androgen synthesis or interfere competitively with the binding of androgens to the AR (Schally et al., 1971). Although a small proportion of patients have undergone tumor-reductive surgery recently  (Wang et al., 2018b; Ranasinghe et al., 2020; Connor et al., 2020), the real results are unclear (Klotz, 2008). Unfortunately, most ADT-sensitive cancers eventually evolve into castration-resistant prostate cancer (CRPC). However, the time to CRPC (TTCRPC) of patients receiving hormonal therapy varies considerably, suggesting that there are significant individual variations in metastatic hormone-sensitive prostate cancer (mHSPC).

Bone is the most common metastasis site in prostate cancer. The prognosis varies widely due to metastases in different bones and the varying numbers of metastasis foci. Bone metastasis risk stratification models that include an extent of disease (EOD) grading system, extremity bone metastases (EBM), and high/low-volume disease (H/LVD) have been proposed (Soloway et al., 1988; Crawford et al., 1989; Gravis et al., 2016; Eisenberger et al., 1998). While these risk stratification models were developed using different clinical endpoints, such as survival rate and disease progression, many investigators demonstrated that these risk stratifications are effective predictors of TTCRPC after primary ADT-based therapy (Kawahara et al., 2020; Mandhani et al., 2016; Lin et al., 2019). However, they exhibit internal heterogeneity and may have poor sensitivity and specificity (Suzuki et al., 2021; Kanesaka et al., 2021; Yamada et al., 2020). Moreover, they do not fully evaluate the impact of different bone metastasis sites (Suzuki et al., 2021), which could be a strong predictor of TTCRPC among patients with mHSPC.

In this study, we established a new bone metastasis risk stratification model by fully exploring the effect of bone metastases at different sites in mHSPC. We also explored the common clinical factors affecting the TTCRPC of patients with mHSPC and used the findings to construct a nomogram.

## Methods

### Patient information

This was a retrospective, single-institution study approved by the ethical committee of the Affiliated Hospital of Xuzhou Medical University (XYFY2022-KL192-01). After review by the ethics committee, it was agreed to conduct the study according to the reviewed study protocol and the informed consent was waived. Patients who were newly diagnosed with *de novo* bone metastatic prostate cancer at our institution from January 2008 to June 2021 were enrolled. The inclusion criteria were: (1) pathologically confirmed prostate adenocarcinoma; (2) complete imaging data and imaging-confirmed bone metastases; (3) no prostate cancer-related treatment prior to pathological diagnosis; and (4) treated with ADT or ADT-based therapy, such as surgical/pharmacological castration+anti-androgen therapy (bicalutamide; flutamide)/androgen biosynthesis inhibitor (abiraterone). The exclusion criteria were: (1) visceral metastasis; (2) adenocarcinoma with neuroendocrine differentiation; (3) the presence of other cancers that affect survival; (4) subsequent tumor-reductive surgical treatment; and (5) incomplete clinicopathologic and follow-up data.

The patients’ demographic and clinicopathological data, such as age, height, weight, hypertension (Hp), diabetes mellitus (DM), prostate-specific antigen (PSA), PSA nadir (nPSA), time to PSA nadir (TTN), alkaline phosphatase (ALP), hemoglobin (Hb), fibrinogen (Fib), clinical T stage [cT], and Gleason score were obtained from their medical records. The tumor-node-metastasis (TNM) stage was assigned according to the recommendation (Buyyounouski et al., 2017). Eventually, a total of 122 patients were included in the study.

### Bone metastatic evaluation

Bone scan images were obtained before therapy. We re-reviewed all bone scans and recorded the metastases sites and numbers. Previous bone-based risk stratification models, *i.e.,* EOD, EBM and H/LVD, have been described previously (Soloway et al., 1988; Gravis et al., 2016; Eisenberger et al., 1998).

Our model was based on a previous classification of bone metastatic sites (Suzuki et al., 2021) and classified metastasis into six sites: skull; cervical, thoracic, and lumbar vertebrae; chest (ribs and sternum); pelvis; upper limbs; and lower limbs. We calculated the bone metastatic score (BMS) for each site as follows: 0 points were assigned for non-metastasis and 1 point was assigned for metastasis. The scores were summed to obtain the BMS (range: 0–6 points). To group patients according to BMS, we used the X-tile software (version 3.6.1) (Camp, Dolled-Filhart & Rimm, 2004) to obtain optimum cutoff values (High-risk group [HRG]: BMS ≥3; Low risk group [LRG]: BMS < 3).

### Follow-up

The patients were followed up mainly by telephone, combined with outpatient and/or inpatient visits. The contents of the follow-up visits were recorded on designated forms. The endpoint was CRPC. The duration was calculated from the date of prostate biopsy. CRPC was defined based on the recommendation (Cornford et al., 2017). The last follow-up date was March 4, 2022 and patients with mHSPC at the last follow-up were censored.

### Statistical methods

The numerical variables and categorical variables were described as medians (interquartile ranges [IQRs]) and numbers (percentages), respectively. The TTCRPC was estimated using the Kaplan–Meier method. Numerical variables were converted into binary variables according to reference range of the normal upper or lower limit and/or previous article classification standards (Lin et al., 2019; Kanesaka et al., 2021; Tian et al., 2020). The Cox proportional hazards regression model was used in the univariable and multivariable analyses to calculate the hazard ratios (HRs). Clinicopathological variables that demonstrated a univariable relationship with the TTCRPC (*p* < 0.100) and were considered clinically relevant were subsequently entered into the multivariable model.

The new bone risk stratification was validated by calculating the area under the receiver operating characteristic (ROC) curve (AUC) using R software to compare the validities of the H/LRG and the other models  (Bouassida et al., 2021). The nomogram for predicting the 1-, 2-, and 3-year CRPC was developed using Cox-derived coefficients. Model calibration and discrimination were assessed with calibration plots and the C-index. The model overfitting was quantified using bootstrapped resampling (1,000).

A two-sided *p* < 0.050 was considered statistically significant. Analyses were performed using Statistical Product and Service Solutions (SPSS, version 26.0; SPSS, Inc., Chicago, IL, USA) and R (version 4.1.1; R Core Team, 2021).

## Results

### Baseline characteristics of the patients

Table 1 lists the study population’s demographic and clinicopathological characteristics. The median patient age was 73 years. Forty-two patients (34.4%) presented with hypertension and/or diabetes. Most patients had Gleason score ≤ 8 (93 cases, 76.2%) and high-volume disease (84 cases, 68.9%). The follow-up period was 2–92 months with a median of 11.5 months. Eighty-six patients progressed to CRPC during the follow-up.

**Table 1:** Patients’ characteristics.

	Number	Percentage	Median (interquartile range)
Age (year)			73 (66–78)
Hp/DM			
NO	80	65.6%	
YES	42	34.4%	
Gleason score			
≤8	93	76.2%	
>8	29	23.8%	
Clinical T stage			
cT1-T2	54	44.3%	
cT3-T4	68	55.7%	
EOD			
1–2	74	60.7%	
3–4	48	39.3%	
High/Low-volume disease			
Low	38	31.1%	
High	84	68.9%	
Extremity bone metastases			
NO	59	48.4%	
YES	63	51.6%	
PSA(ng/ml)			100.00 (72.37–397.95)
nPSA(ng/ml)			1.40 (0.12–8.27)
TTN(months)			4 (2–8)
Hb(g/L)			126.50 (114.00–137.25)
ALP(U/L)			119.50 (74.50–297.25)
Fib(g/L)			3.91 (2.97–4.62)

**Notes.**

DM diabetes mellitus Hp hypertension PSA prostate-specific antigen nPSA prostate-specific antigen nadir TTN time to prostate-specific antigen nadir Hb hemoglobin ALP alkaline phosphatase Fib fibrinogen

### Establishment of bone risk stratification model

The most common bone metastatic site was the pelvis (90.2%), followed by the cervical, thoracic, or lumbar vertebrae (69.7%), chest (68.0%), upper limbs (56.6%), lower limbs (49.2%) and skull (28.7%) (Table 2). Univariable Cox analysis revealed that almost every metastatic site (except the pelvis) was an important factor affecting TTCRPC (*p* < 0.05) (Table 2). The median BMS was 4 (Table 3), where approximately 50% of the patients had scores of ≥4. According to the H/LRG criteria, 76 patients had HRG, while 46 had LRG.

**Table 2:** Univariable Cox regression results of bone metastasis.

		Number = 122	Percentage	TTCRPC		
				HR	95% CI	*P*
Skull	NO	87	71.3%	1		
	YES	35	28.7%	1.831	1.155–2.901	0.010
Cervical/Thoracic/Lumber vertebrae	NO	37	30.3%	1		
	YES	85	69.7%	2.260	1.3440–3.812	0.002
Pelvis	NO	12	9.8%	1		
	YES	110	90.2%	1.827	0.840–3.973	0.129
Chest (rib and sternum)	NO	39	32.0%	1		
	YES	83	68.0%	2.093	1.272–3.444	0.004
Upper limbs	NO	53	43.4%	1		
	YES	69	56.6%	2.729	1.712–4.348	<0.001
Lower limbs	NO	62	50.8%	1		
	YES	60	49.2%	1.987	1.283–3.078	0.002

**Table 3:** Bone metastatic score.

Score	Number	Percentage
1	27	22.1%
2	19	15.6%
3	11	9.0%
4	13	10.7%
5	20	16.4%
6	32	26.2%

**Figure 1:**
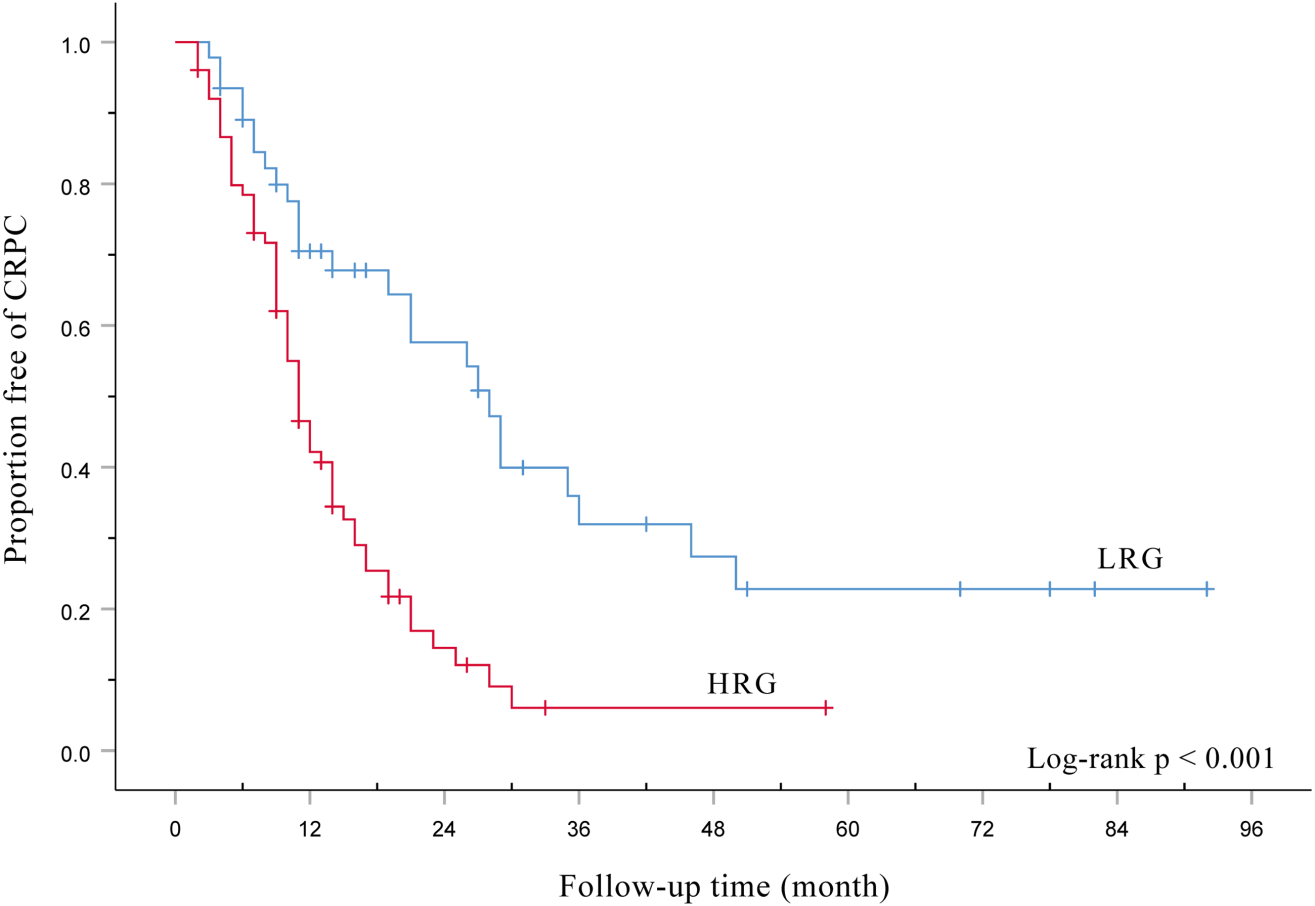
Kaplan-Meier analysis of the time to CRPC according to the H/LRG criteria.

**Figure 2:**
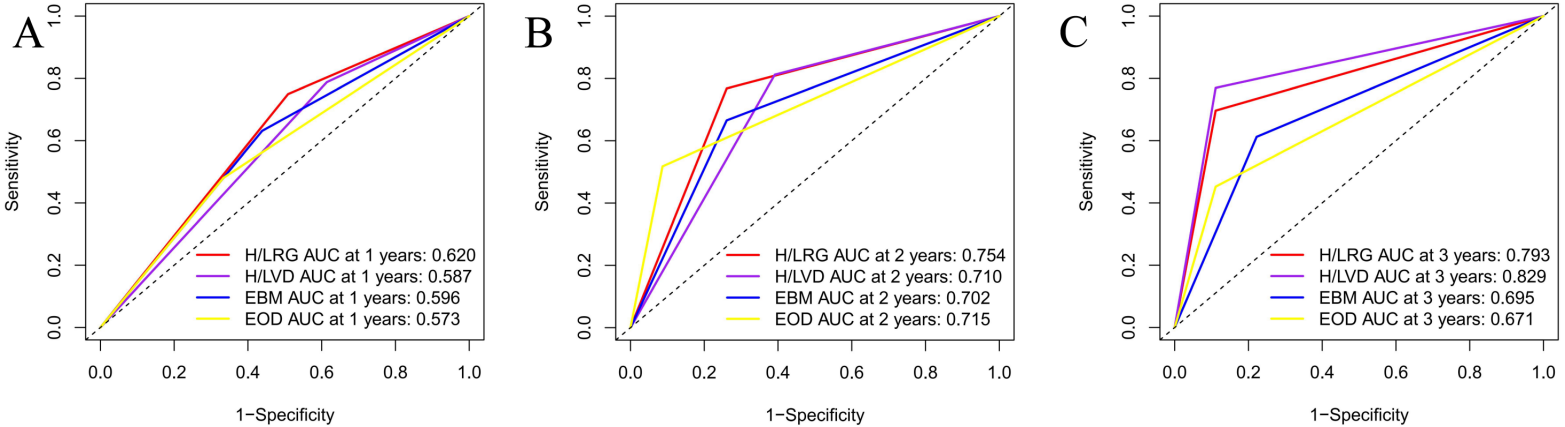
Comparison of AUC analysis of each risk stratification.

Figure 1 shows HRG had a signifcantly poorer TTCRPC than the LRG(*p* < 0.001). Figure 2 displays the comparison of our model with the other three risk stratification models (EOD, EBM, and H/LVD) according to their CRPC AUCs. The 1-, 2-, and 3-year AUCs for the EOD, H/LVD, and EBM were 0.573, 0.715, and 0.671; 0.587, 0.710, and 0.829; and 0.596, 0.702, and 0.695, respectively. The 1-, 2-, and 3-year AUCs for our model were 0.620, 0.754, and 0.793, respectively. H/LRG demonstrated higher validity than the other risk stratification models.

### Prognostic factors for CRPC

In the univariable analysis, nPSA (>0.2 ng/ml *vs.* ≤0.2 ng/ml; HR 3.778, 95% CI [2.154–6.627], *p* < 0.001), H/LRG (HRG *vs.* LRG; HR 2.644, 95% CI [1.623–4.307], *p* < 0.001), TTN (≤6 months *vs.* >6 months; HR 3.158, 95% CI [1.888–5.281], *p* < 0.001), ALP (>128 U/L *vs.* ≤128 U/L; HR 2.335, 95% CI [1.512–3.605], *p* < 0.001), and Gleason score (>8 *vs.* 8; HR 1.676, 95% CI [1.044–2.689], *p* = 0.032) demonstrated statistically significant effects on TTCRPC (Table 4).

Considering that ALP demonstrated collinearity with bone metastases, ALP was not included in the multivariable analysis. Finally, five variables (Gleason score, H/LRG, nPSA, TTN, and Fib) were included in a multivariable Cox proportional hazard regression model for the analysis. The results demonstrated that Gleason score (>8 *vs.* ≤8; HR 1.860, 95% CI [1.125–3.075], *p* = 0.016), nPSA (≤0.2 ng/ml *vs.* >0.2 ng/ml; HR 2.384, 95% CI [1.242–4.573], *p* = 0.009), TTN (≤6 months *vs.* >6 months; HR 2.243, 95% CI [1.257–4.003], *p* = 0.006), and H/LRG (HRG *vs.* LRG; HR 1.864, 95% CI [1.108–3.135], *p* = 0.019) were independent factors influencing the TTCRPC (Table 4).

### Development and validation of nomogram for predicting CRPC

**Figure 3:**
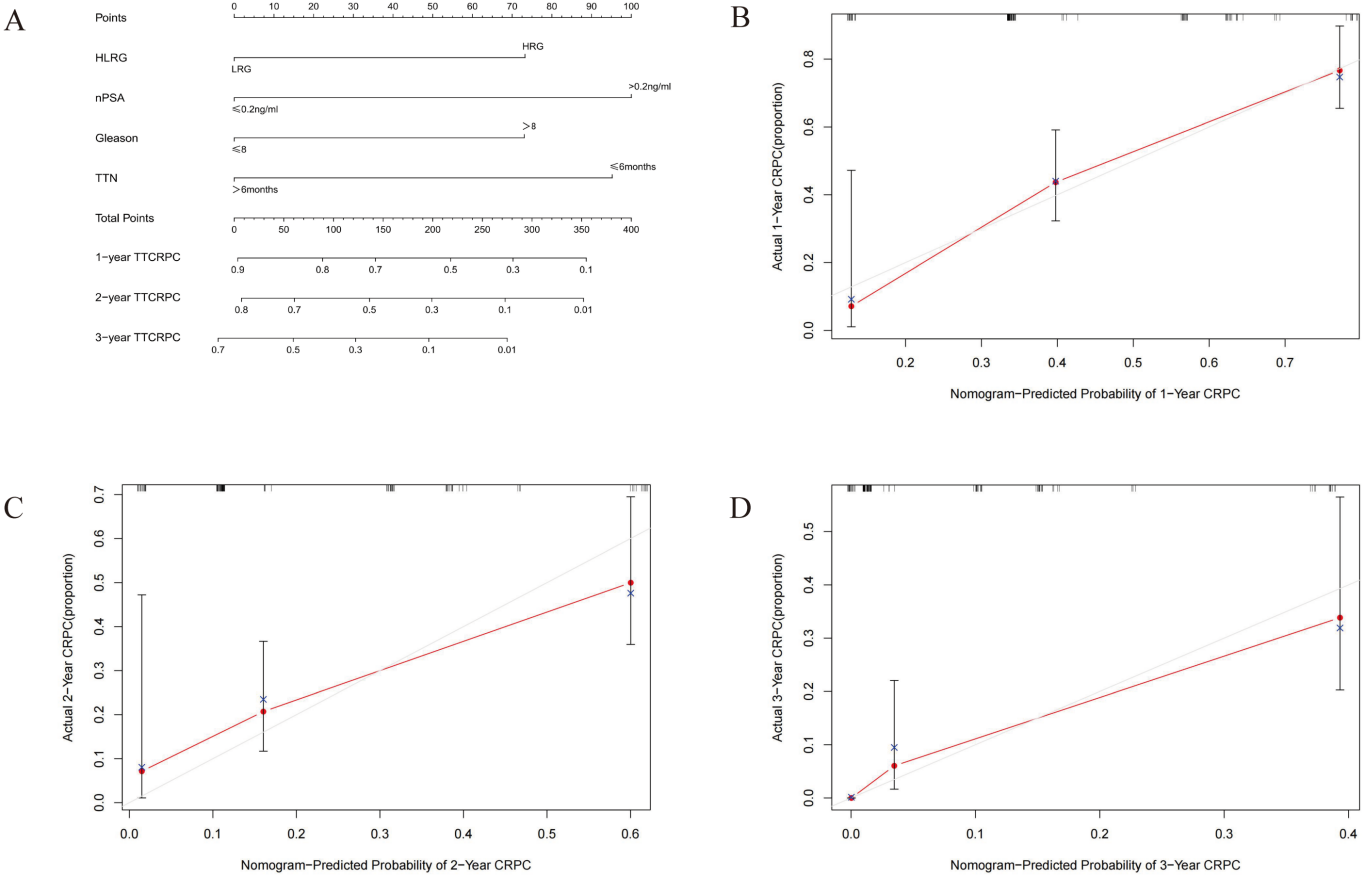
(A–D) Nomogram and calibration curve for the prognostic prediction model.

We developed a nomogram based on four parameters (Gleason score, H/LRG, nPSA, and TTN) to predict the 1-, 2-, and 3-year CRPC (Fig. 3). The effectiveness of the model was examined with discrimination evaluation (C-index) and conformity assessment (calibration curve plotting). The C-index was calibrated during the internal validation in R using the bootstrap method, and the 1-, 2-, and 3-year calibration curves were plotted. With the bootstrap = 1,000 setting, the evaluation results demonstrated that the CRPC nomogram prediction model had a C-index of 0.727, indicating that the CRPC nomogram prediction model had good predictive accuracy. Accordingly, the calibration curve of the model was plotted (Fig. 3), and demonstrated that the model had good conformity.

**Table 4:** Univariable and multivariable analyses of factors associated with time to CRPC.

Time to CRPC	Univariable				Multivariable			
			95% Cl				95% Cl	
Variables	*P* value	HR	Lower	Upper	*P* value	HR	Lower	Upper
Age > 65 *vs.*≤65 (years)	0.807	1.066	0.640	1.775	–	–	–	–
Hp/DM Yes *vs.* No	0.548	0.870	0.551	1.372	–	–	–	–
PSA > 100 *vs.*≤100 (ng/mL)	0.627	1.120	0.708	1.771	–	–	–	–
nPSA > 0.2 *vs.*≤0.2 (ng/mL)	<0.001	3.778	2.154	6.627	0.009	2.384	1.242	4.573
TTN ≤ 6 *vs.*>6 (months)	<0.001	3.158	1.888	5.281	0.006	2.243	1.257	4.003
Hb < 130 *vs.*≥130 (g/L)	0.533	1.147	0.746	1.764	–	–	–	–
ALP > 128 *vs.*≤128 (U/L)	<0.001	2.335	1.512	3.605	–	–	–	–
Fib > 4 *vs.*≤4 (g/L)	0.082	1.456	0.953	2.225	0.967	1.009	0.654	1.557
Gleason score > 8 *vs.*≤8	0.032	1.676	1.044	2.689	0.016	1.860	1.125	3.075
Clinical T stage > 2 *vs.*≤2	0.734	1.077	0.701	1.656	–	–	–	–
HRG *vs.* LRG	< 0.001	2.644	1.623	4.307	0.019	1.864	1.108	3.135

**Notes.**

DM diabetes mellitus Hp hypertension PSA prostate-specific antigen nPSA prostate-specific antigen nadir TTN time to prostate-specific antigen nadir Hb hemoglobin ALP alkaline phosphatase Fib fibrinogen HRG high-risk group LRG low-risk group

## Discussion

In this study, we established a novel H/LRG risk stratification for mHSPC using whole-body bone imaging data. The 1-, 2-, and 3-year CRPC AUCs of our model were 0.620, 0.754, and 0.793, respectively. A nomogram based on four parameters (Gleason score, H/LRG, nPSA, and time to PSA nadir) was developed to predict the TTCRPC.

Primary ADT has been the standard of care for mHSPC over 50 years. Bone is the most common metastasis site for patients with mHSPC, and the prognosis varies widely among cases, with metastases to different bones and different numbers of metastatic foci. Previous researchers have proposed risk stratification methods based on whole-body bone imaging, including EOD scores, EBM, and H/LVD. Although these methods yielded important prognostic information, they were unable to fully evaluate the impact of different bone metastasis sites on prognosis. Furthermore, the EOD score and H/LVD are both subjective and semi-quantitative parameters (Miyoshi et al., 2017). Accurate evaluation of bone metastases would lead to an appropriate prediction of CRPC, which would be valuable for patient counseling.

Prostate cancer metastasizes by following the venous drainage system through the lower paravertebral plexus (Batson’s plexus), which is why the pelvis and spine are the most common metastasis sites (Bubendorf et al., 2000). Therefore, bone metastases in the extremities are often at an advanced disease stage and are considered a marker of poor overall survival. However, few investigators explored the effect of bone metastases at different sites on the TTCRPC. Based on the classification of bone metastasis sites in previous studies, we classified the metastatic sites into six sites: a combination of both qualitative and semi-quantitative methods. Interestingly, the bone metastases at the six sites had a similar effect on prognosis (Table 2). Therefore, we assigned a score of 0 or 1 point to each site based on whether these areas had metastases (range 0–6). We used X-tile to acquire optimal cutoff points of BMS. Then, we defined HRG as BMS ≥3 and LRG as BMS < 3.Finally, we analyzed four risk stratification models according to their CRPC AUCs and found that the newly constructed risk stratification demonstrated higher specificity and sensitivity compared with other risk stratifications. In the multivariable analysis, H/LRG remained an independent prognostic factor for TTCRPC (Table 4). The major strength of this study is that the novel risk stratification fully evaluated the prognostic impact of bone metastases at various sites. Furthermore, the re-reviewed method for assessing bone metastases relatively reduced the subjective errors.

Previously, indicators such as PSA, Gleason score, and cT were considered important prognostic indicators of TTCRPC and several studies recently confirmed the association of nPSA and TTN with prostate cancer prognosis (Tian et al., 2020; Akamatsu et al., 2019; Ali et al., 2021; Wang et al., 2018a). In this study, we found that a longer TTN indicated a better TTCRPC for patients with mHSPC. Intuitively, most clinicians tend to think that a more rapid PSA decline in response to primary ADT and a shorter TTN would be positively associated with favorable CRPC (Sasaki & Sugimura, 2018). However, the relationship between TTN and TTCRPC remains an unsettled debate with contradictory findings (Hori et al., 2011). In accordance with our findings, several studies demonstrated that longer TTN after primary ADT predicted favorable TTCRPC in mHSPC (Hori et al., 2011; Morote et al., 2004; Huang et al., 2011). In contrast, Oefelein et al. (2002) indicated that a longer TTN predicted a worse prognosis (Hori et al., 2011). Besides, there is no consensus on the exact TTN , with studies reporting different thresholds.

Nomograms represent useful tools for estimating the prognosis for various cancers (Qiu et al., 2022; Xie et al., 2022). In this study, we developed a nomogram based on four parameters (Gleason score, nPSA, TTN, and H/LRG) to predict the TTCRPC. Calibration plots and the C-index demonstrated that the model performed well. Therefore, the novel risk stratification and nomogram model may be a useful tool for clinical doctors to evaluate patient prognosis.

Nonetheless, our study has limitations that should be emphasized. First, it was a retrospective study, which has inherent limitations. Second, the patients had various health statuses and we could not fully assess their physical conditions, which may have affected mHSPC treatment outcomes. Finally, this was a single-center study with a relatively small sample size, and multicenter studies with large sample sizes are needed to confirm our findings and validate the accuracy of the newly developed model.

## Conclusion

In this study, we established a novel H/LRG risk stratification model and nomogram for newly diagnosed *de novo* bone metastatic prostate cancer. Internal validation determined that the prognostic nomogram model based on the Gleason score, H/LRG, nPSA, and TTN was effective as a prognostic predictor of ADT based therapy response with good accuracy in patients with mHSPC. The model can provide a reference for clinicians to assess the prognosis of metastatic prostate cancer and be a valuable clinical tool.

##  Supplemental Information

10.7717/peerj.14615/supp-1 Data S1 Raw data

## Additional Information and Declarations

### Data Availability

The following information was supplied regarding data availability: The raw data are available in the Supplemental File.
